# Sympathetic-mediated blunting of forearm vasodilation is similar between young men and women

**DOI:** 10.1186/s13293-022-00444-0

**Published:** 2022-06-25

**Authors:** Alessandro Gentilin, Paolo Moghetti, Antonio Cevese, Federico Schena, Cantor Tarperi

**Affiliations:** 1 Department of Neuroscience, Biomedicine, and Movement Sciences, University of Verona, Verona, Italy; 2 Italian Institute for Cardiovascular Research (INRC), Bologna, Italy; 3 Section of Endocrinology, Diabetes and Metabolism, Department of Medicine, University of Verona and Azienda Ospedaliera Universitaria Integrata Verona, Verona, Italy; 4 Department of Clinical and Biological Sciences, University of Turin, Turin, Italy

**Keywords:** Endothelium, Rapid vasodilation, Cold pressor test

## Abstract

**Background:**

The in-vivo regulation of vascular conductance (VC) is a continuous balance between endothelial vasodilation and sympathetic vasoconstriction. Although women may report blunted sympathetic vasoconstriction along with higher endothelial vasodilation than men, it is currently unknown whether the interaction between vasoconstriction and vasodilation leads to different regulation of VC between sexes. This study assessed sex differences in sympathetic-mediated blunting of endothelial vasodilation after a brief period of ischemia and whether any restriction of vasodilation blunts tissue blood flow (BF) and re-oxygenation.

**Methods:**

13 young women and 12 young men underwent two 5-min forearm circulatory occlusions followed by reperfusion, one in basal conditions and the other during cold pressor test-induced sympathetic activation (SYMP). Brachial artery diameter and BF, mean arterial pressure, total peripheral resistance (TPR), and thenar eminence oxygenation were collected. Percent changes normalized to baseline values of forearm VC, brachial artery BF and flow-mediated dilation (FMD), TPR, and hand oxygenation after circulatory reperfusion were calculated.

**Results:**

TPR increased during SYMP in men (*p* = 0.019) but not in women (*p* = 0.967). Women showed a greater brachial artery FMD than men (*p* = 0.004) at rest, but sex differences disappeared after normalization to shear rate and baseline diameter (*p* > 0.11). The percent increases from baseline of peak and average forearm VC after circulatory reperfusion did not differ between sexes in basal conditions (*p* > 0.98) or during SYMP (*p* > 0.97), and were restrained by SYMP similarly in both sexes (*p* < 0.003) without impairing the hand re-oxygenation (*p* > 0.08) or average hyperemic response (*p* > 0.09).

**Conclusions:**

Although women may report blunted sympathetic vasoconstriction than men when assessed separately, the similar sympathetic-mediated restriction of vasodilation suggests a similar dynamic regulation of VC between sexes. SYMP-mediated restrictions of the normal forearm vasodilation do not impair the average hyperemic response and hand re-oxygenation in both sexes.

**Supplementary Information:**

The online version contains supplementary material available at 10.1186/s13293-022-00444-0.

## Introduction

Sex differences in sympathetic neurovascular regulation have been described [1–3]. In young individuals, muscle sympathetic nerve activity (MSNA) appears to be lower in women compared to men at rest [2]. MSNA seems to be correlated to peripheral vascular resistance in young men, but not in young women [2–4]. Sympathetic stimulants have been shown to induce less vasoconstriction in women compared to men despite similar MSNA increments [2]. The previous findings support the notion that the transduction of sympathetic activity into vascular resistance is blunted in young women [2–4]. Sympathetic neurovascular transduction has been proposed to differ between sexes due to multiple factors, including a different sensitivity and distribution of post-junctional α-adrenergic and β-adrenergic receptors [1, 5]. It has been postulated that β-adrenergic vasodilator mechanisms offset α-adrenergic vasoconstriction in women compared to men [6]. When β-adrenergic (vasodilation) activity was blocked, sex differences in resting vasoconstriction responsiveness were abolished [5]. Differences in the type and quantity of neurotransmitters released from the sympathetic nervous system have also been suggested to account for sex differences in neurovascular modulation [7]. Interestingly, as excellently described by Hissen et al. [3], the previous findings supporting a blunted vascular transduction of sympathetic activity in women are not universal and strongly depend on the approach used to assess vascular transduction. When sex differences are investigated on a beat-by-beat basis at rest, the preponderance of the previous studies has suggested similar levels of sympathetic vascular transduction between young men and women [3]. Women also seem to have higher endothelial-mediated vasodilation compared to men as suggested by their greater artery flow-mediated vasodilation (FMD) [8]. A key role for oestrogens in relaxing vascular smooth muscle has been suggested in women [9]. The previous research, however, has investigated sex differences in vasodilator and vasoconstrictor responses separately. This approach does not define how these controls interact and how the final neurovascular regulation differs between sexes in controlling vascular conductance (VC) and blood flow (BF) in-vivo. The in-vivo regulation of VC is indeed a dynamic process, involving the continuous balance between sympathetic vasoconstriction and endothelial-mediated vasodilation [10]. The fine regulation of VC is essential for regulating tissue BF and systemic blood pressure [11].

Sudden vasodilation of a vascular tissue following a period of ischemia provides a fast blood supply to an area in need of oxygen [12]. This takes on particular importance, where oxygen-requiring tissues are vital. Considering the remarkable difference between sexes in the prevalence and harshness of cardiovascular disease [1, 7, 9], the identification of sex differences in neurovascular modulation is pertinent as these may provide new insights into cardiovascular medicine. The identification of factors that affect vascular health in one sex can lead to the development of translational studies and therapies to be applied to the other sex, as well as to a differentiated treatment between sexes [13]. This study aims to identify sex differences in the acute sympathetic activation (SYMP)-mediated blunting of forearm vasodilation after a brief period of ischemia to assess the interaction between sympathetic vasoconstriction and endothelial-mediated vasodilation in both sexes. In addition, this study aims to investigate whether any SYMP-mediated restriction of vasodilation blunts tissue blood flow (BF) and re-oxygenation after circulatory reperfusion in both sexes. According to the current literature, women may have lower sympathetic vasoconstriction as well as higher endothelial-mediated vasodilation compared to men [7]. Therefore, it seems reasonable to hypothesize that any attenuation of vasodilation via SYMP is lower in women compared to men. Moreover, it is hypothesized that SYMP impairs the normal tissue BF and re-oxygenation after circulatory reperfusion in both sexes, but to a greater extent in men compared to women.

## Methods

### Participants

25 young, healthy, non-smoker, recreationally active individuals were recruited for this study (Table 1). There were 12 men and 13 women. Participants met the inclusion (absence of any muscle–skeletal, metabolic, cardiovascular, and respiratory disease; between 18 and 25 years of age) and exclusion (BMI ≥ 28 kg/m^2^; diabetes mellitus; hypertensive disorders; use of any drug altering the cardiovascular response to SYMP; family history of premature cardiovascular disease) criteria. Women were not on contraceptives and were tested during the early follicular phase (days 1–7) of the menstrual cycle, according to the current recommendations for the assessment of FMD in humans [14, 15]. This phase offers the lowest attainable levels of estrogen and progesterone, in which hormone levels and artery FMD in women are comparable to those of men [16]. All experiments were performed in the morning (at around 10.00 AM) in a quiet and temperature-controlled room (~ 22 °C). Participants were fasting and were asked to abstain from alcohol and caffeine in the 48 h before the tests. The number of subjects was calculated with an a priori power analysis (GPower 3.1.9.7; Universität Düsseldorf, Germany) for an *F* test (ANOVA, repeated measures, within-between interaction), partial eta squared of 0.20, statistical power (1 − *β*) of 0.80, level of significance of 0.05. This analysis suggested the need for 7 men and 7 women to assess the sympathetic-mediated blunting of vasodilation. The effect size was calculated according to the different magnitude of sympathetic-mediated restriction of leg VC between sexes in response to the cold pressor test (CPT) [17]. The Ethics Board of the University of Verona approved all procedures involving human subjects (3293CESC). Each participant provided written informed consent before being involved in any test.

**Table 1:** Characteristics of subjects involved in the study

	Men (*n* = 12)	Women (*n* = 13)	Men vs Women (*p* value)
Age (years)	25.6 ± 3.7	23.8 ± 2.6	0.04
Height (cm)	173.6 ± 5.5	163.8 ± 5.5	0.0002
Weight (kg)	75.92 ± 5.16	56.77 ± 5.05	< 0.0001
Baseline mean arterial pressure (mmHg)	79.9 ± 7.6	75.9 ± 6.3	0.17
Baseline brachial artery diameter (cm)	0.39 ± 0.07	0.31 ± 0.04	0.002
Baseline brachial blood flow (mL/min)	85.3 ± 32.9	49.9 ± 26.9	0.01
Baseline vascular conductance (mL/min/mmHg)	1.04 ± 0.47	0.66 ± 0.35	0.03
Baseline heart rate (bpm)	67.5 ± 6.8	66.0 ± 16.1	0.85

### Subject monitoring

Participants lay supine with their knees bent over the rim of the bed during the experiment. Their right arm was extended for ultrasound measurements. Subjects were fitted with an automatic blood pressure monitor (Tango+, SunTech Medical, Morrisville, NC, USA) at the heart level on their left arm. Subjects were also equipped with a beat-by-beat finger blood pressure monitoring system (Portapres; Finapres Medical System BV, The Netherlands) on their left hand to measure the mean arterial pressure (MAP). The beat-by-beat finger blood pressure system was calibrated with the automatic sphygmomanometer recording the brachial blood pressure. Participants were also instrumented with the 3-lead electrocardiograph (ECG) of the Ultrasound Device (LOGIQ S7 pro, GE, Milwaukee, USA). Near-infrared spectroscopy (NIRS; OxiplexTS, ISS, USA) was used to assess hand oxygenation. NIRS probe was placed on the thenar eminence of the right hand and was completely covered to ensure that environmental light could not reach the probe. A pressure cuff was placed around the right forearm, distal to the imaged artery, 2 cm below the flexion point of the elbow. The cuff was inflated with a rapid cuff inflator (Hokanson, Bellevue, USA) > 50 mmHg above the systolic blood pressure. The cuff could be deflated within approximately 300 ms. While subjects were asked to stay relaxed and breathe regularly, the right brachial artery was scanned via pulsed Doppler ultrasonography to simultaneously detect mean blood velocity and measure the brachial artery diameter. The probe location was marked to evaluate the same artery section at rest and during SYMP. Data were collected using a 4.4 MHz probe with a 60° angle of insonation. The ultrasound gate was adjusted to examine the whole artery width. The sample volume was aligned and regulated according to vessel size as indicated by recommendations. Brachial artery FMD was measured above the antecubital fossa. Ultrasound measures were performed by an expert sonographer with > 500 h of experience. Data were synchronized throughout the experiment by the use of markers.

### Experimental protocol

The right forearm vasodilation was assessed after the release of circulatory forearm occlusion at rest (without SYMP) and SYMP conditions. After 30 min of supine resting, the brachial cuff was inflated for 5 min and then released to induce forearm vasodilation. After an additional 30 min of supine rest, the same procedure was repeated during SYMP. A previous study suggested that FMD assessment could be performed several times with almost identical results with 30 min of rest in between [18]. In accordance with the methodology and timing used in the study by Dyson et al. [19], SYMP was induced by CPT by dipping subjects’ feet in an ice–water slurry (5 °C). The cuff inflation was initiated 2 min after feet submersion in the slurry. The stimulus continued until the conclusion of the experiment. Data were gathered at baseline for 1 min, during the entire occlusion time (5 min), and after cuff release (3 min).

### Vascular data

The forearm VC was calculated by measuring MAP and BF through the brachial artery. Ultrasound data (video clips) were downloaded from the Ultrasound device and analyzed via software (Medical Imaging Applications LLC, USA). The software provided automatic detection of the artery edges along with the mean blood velocity calculation. The diameter was measured every cardiac cycle at the onset of R-waves. Portapres data were exported through its proprietary software (BeatScope 1.1; Finapres Medical System BV, The Netherlands). Ultrasound data (brachial artery diameter, mean blood velocity) and MAP data were analyzed beat-by-beat with a 3-beat rolling average. Then, data were fitted to avoid erroneous calculations of peak forearm VC or peak brachial artery FMD [19], as well as to extrapolate second-by-second data. Brachial artery BF was calculated as mean blood velocity (cm*s^−1^)*π*r^2^*60 (ml*min^−1^), where *r* is the radius of the brachial artery. Forearm VC was calculated as brachial artery BF divided by MAP.

The primary research outcome was the percent increase from preceding baseline values of forearm VC upon cuff release. We calculated the percent increase from baseline of peak forearm VC after cuff release as well as the percent increase from baseline of the average forearm VC over the 60 s following cuff release. We also calculated the difference of such percent increments in forearm VC between rest and SYMP (delta value: percent increase at rest minus percent increase at SYMP), to quantify the extent to which SYMP restrains the normal increase in VC in both sexes. Similar calculations were repeated for brachial artery BF data. The brachial artery FMD was an additional research outcome. Brachial artery peak FMD was also calculated as the percent increase from baseline after cuff release [14, 15]. Shear rate was determined as four times mean blood velocity divided by artery diameter. The peak value of shear rate upon cuff release was identified. The cumulative shear rate was calculated as the area under the curve (AUC) from cuff release to the peak brachial artery diameter. Brachial artery FMD was normalized to cumulative shear rate and baseline diameter. In addition, allometric scaling of FMD was also calculated as previously indicated [20] to control for statistical bias towards the brachial artery baseline diameter.

### Hemodynamic data

Beat-by-beat cardiac output, stroke volume, MAP, total peripheral resistance (TPR), and heart rate were non-invasively calculated through the Modelflow algorithm of Portapres. These data were recorded on the left hand to obtain information on systemic changes before, during, and after circulatory occlusion at rest and during SYMP. Hemodynamic data were averaged over the 60 s after cuff release, in concomitance with the vascular data collection, in both rest and SYMP conditions. The percent changes from the previous baseline values (pre-cuff inflation) of all hemodynamic data after cuff release were calculated.

### NIRS data

The average oxygen saturation of the thenar eminence was calculated at baseline, over the 60 s before cuff release, and over the 60 s following cuff release. Values of oxygen saturation during ischemia and after cuff release are reported as percent changes from the preceding baseline values.

### Statistics

Statistical comparison was performed on the data collected at rest vs during SYMP in men vs women. Data normality was tested with the Shapiro–Wilk normality test. A two-way repeated-measure ANOVA with a Sidak posthoc test was used to assess any effects of SYMP and sex on the forearm VC, brachial artery BF, hemodynamic data, and hand oxygenation. GraphPad Prism 8 (GraphPad Software, San Diego, United States) was used for statistical analysis and graphs. The analysis of covariance required for the allometric scaling of the brachial artery FMD was performed with MATLAB (MathWorks, USA). Results are expressed as mean ± standard deviations.

## Results

All data passed the normality test. ANOVA results are reported in Table 2. Numerical data and effect sizes are also provided in the Additional file 1.

**Table 2:** The table reports the ANOVA results

	Effect of sex (*p* value)	Effect of SYMP (*p* value)	Interaction (*p* value)
Peak forearm VC change (%)	0.97	< 0.0001	0.42
Mean forearm VC change (%)	0.84	< 0.0001	0.94
Peak brachial artery BF change (%)	0.95	< 0.0001	0.25
Mean brachial artery BF change (%)	0.73	0.03	0.54
Hand oxygenation after cuff inflation (%)	0.70	0.16	0.54
Hand oxygenation after cuff release (%)	0.004	0.009	0.80
Brachial artery FMD (%)	0.03	< 0.0001	0.03
FMD normalized to baseline diameter (% × cm)	0.25	< 0.0001	0.11
FMD normalized to cumulative shear stress (e^−005^%/s^−1^ × 60 s)	0.08	< 0.0001	0.34
TPR change (%)	0.04	0.01	0.03
MAP change (%)	0.41	< 0.0001	0.43
Heart rate change (%)	0.98	< 0.0001	0.83
Cardiac output change (%)	0.75	0.50	0.55
Cardiac stroke volume change (%)	0.22	0.23	0.85

### Vascular conductance

**Fig. 1:**
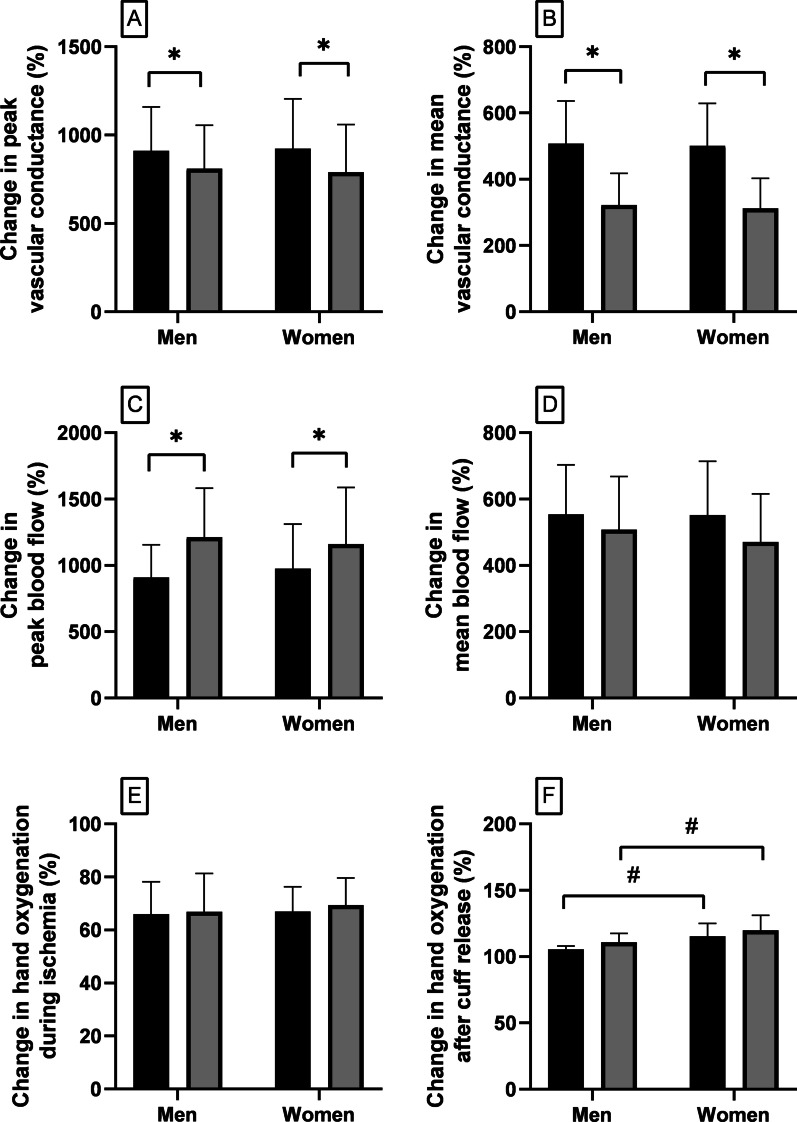
Percent change from preceding baseline values in peak (**A**) and mean (**B**) vascular conductance after cuff release, peak (**C**) and mean (**D**) blood flow after cuff release, hand oxygenation during ischemia (**E**), and hand oxygenation after cuff release (**F**), at rest (black bars) vs SYMP (grey bars) (*p < 0.05), in men vs women (^#^*p* < 0.05)

Baseline forearm VC (Table 1) was lower in women compared to men (*p* = 0.03). The percent increase from baseline of peak forearm VC after cuff release (Fig. 1A) did not differ between sexes either at rest (*p* = 0.99) or during SYMP (*p* = 0.98) and was blunted by SYMP in both sexes (*p* < 0.003). The percent increase from baseline of the average forearm VC over the 60 s after cuff release (Fig. 1B) did not differ between sexes either at rest (*p* = 0.98) or during SYMP (*p* = 0.97) and was blunted by SYMP in both sexes (*p* < 0.0001). Therefore, SYMP restrained the peak (Fig. 3A; *p* = 0.42) and average (Fig. 3B; *p* = 0.94) forearm VC increments to a similar degree in men compared to women. Absolute values of forearm VC after cuff release are reported in the Additional file 1 (Tables S2, S4).

### Blood flow

Baseline brachial artery BF (Table 1) was lower in women compared to men (*p* = 0.01). The percent increase from baseline of peak BF after cuff release (Fig. 1C) did not differ between sexes at rest (*p* = 0.87) or during SYMP (*p* = 0.92), but was increased by SYMP in both sexes (men: *p* = 0.0007; women: *p* = 0.03). The percent increase from baseline of the average BF over the 60 s after cuff release (Fig. 1D) did not differ between sexes at rest (*p* = 0.99) or during SYMP (*p* = 0.80), and was unaffected by SYMP in both sexes (men: *p* = 0.45; women: *p* = 0.09). Therefore, SYMP augmented the normal peak BF (Fig. 3C; *p* = 0.25) and restrained the average BF (Fig. 3D; *p* = 0.54) to a similar degree in men compared to women. Absolute values of BF after cuff release are reported in the Additional file 1 (Tables S6, S8). Absolute values of peak BF resulted to be statistically augmented in men (*p* = 0.004) but not in women (*p* = 0.46) during SYMP compared to at rest.

### Hand oxygenation

Compared to the previous baseline values (100%), the values of hand oxygenation reached in the minute prior to the cuff opening (Fig. 1E) were similar in men compared to women, both at rest (*p* = 0.97) and during SYMP (*p* = 0.83), and were not affected by SYMP (men: *p* = 0.82; women: *p* = 0.28). The average hand oxygenation over the 60 s after cuff release (Fig. 1F) reached values above the preceding baseline in both sexes. However, the values of hand oxygenation reached after cuff release were higher in women compared to men both at rest (*p* = 0.01) and during SYMP (*p* = 0.02), whereas they were not affected by SYMP in both sexes (men: *p* = 0.08; women: *p* = 0.14).

### Brachial artery FMD

**Fig. 2:**
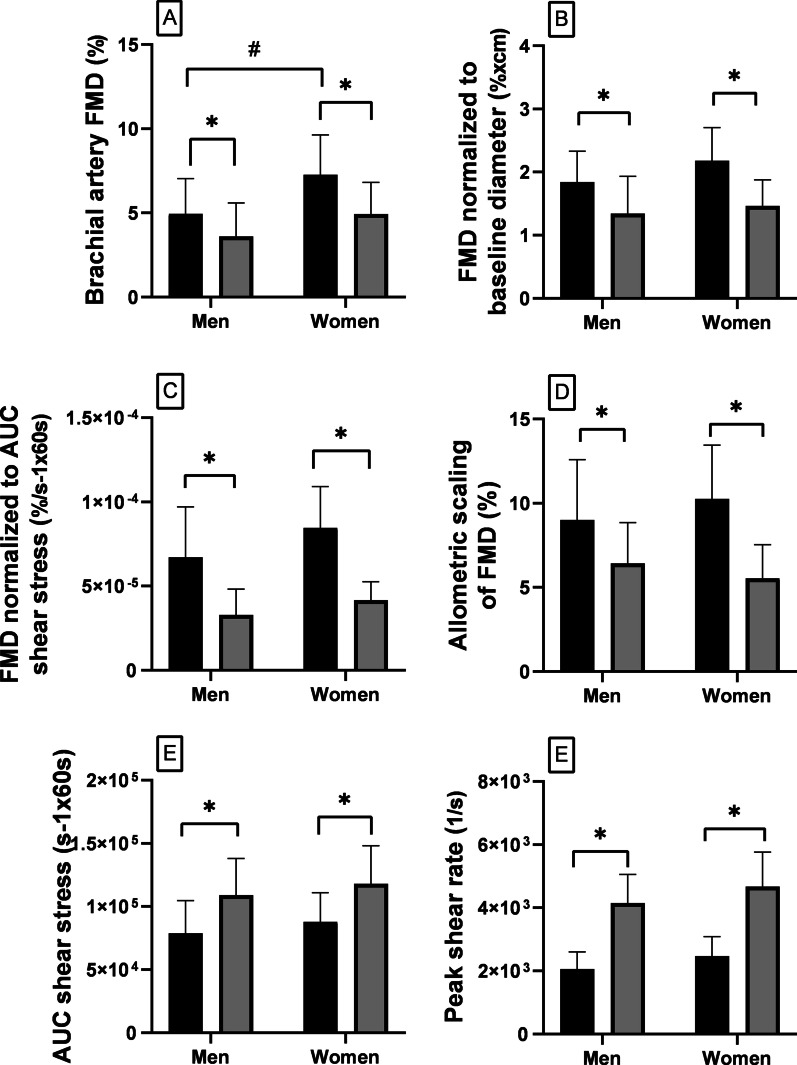
Changes after cuff release in brachial artery flow-mediated dilation (FMD) prior to (**A**) and after normalization to baseline diameter (**B**) and cumulative shear stress (**C**), allometric scaling of brachial artery FMD (**D**), cumulative shear stress (**E**), and peak shear rate (**F**), at rest (black bars) vs SYMP (grey bars) (*p < 0.05), in men vs women (^#^*p* < 0.05)

**Fig. 3:**
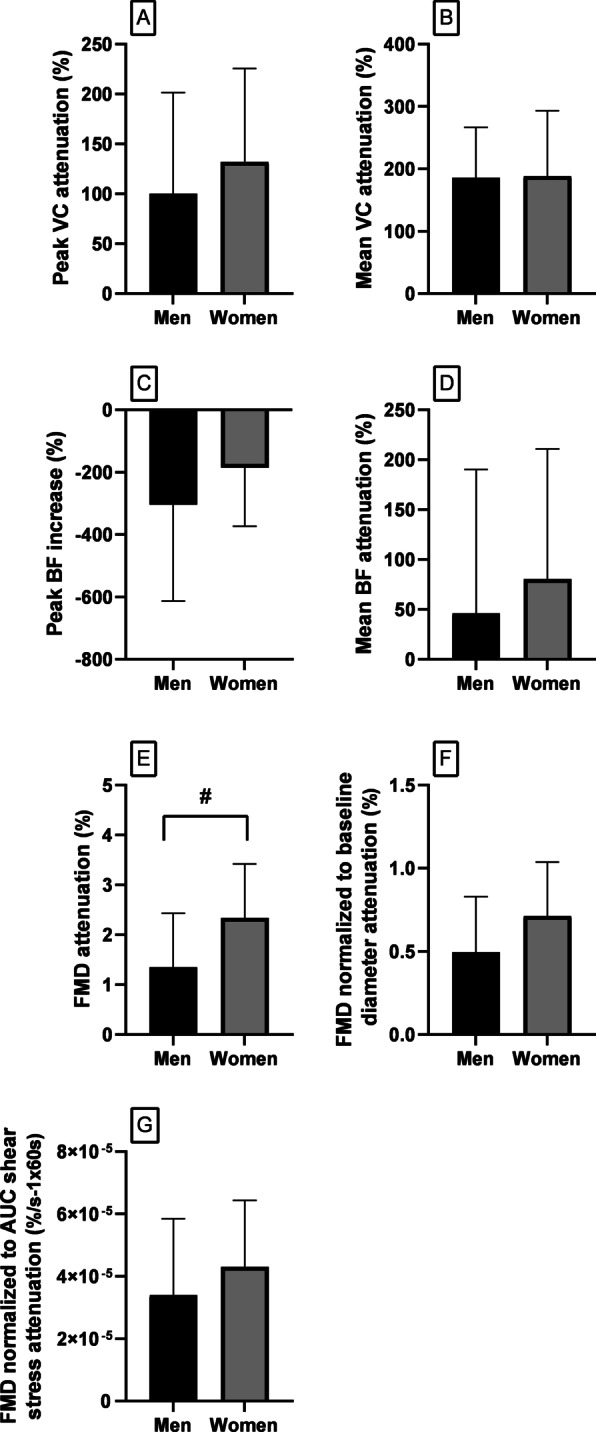
Delta changes (percent increase from baseline at rest minus at SYMP) in peak (**A**) and mean (**B**) vascular conductance, and peak (**C**) and mean (**D**) blood flow, brachial artery flow-mediated vasodilation prior to (**E**) and after normalization to baseline diameter (**F**) and cumulative shear stress (G) (^#^*p* < 0.05)

Baseline brachial artery diameter (Table 1) was lower in women than in men (*p* = 0.002). Prior to performing any normalization, brachial artery FMD (Fig. 2A) was higher in women than men at rest (*p* = 0.016), whereas it was similar during SYMP (*p* = 0.23). FMD normalized to baseline diameter (Fig. 2B) at rest (*p* = 0.20) and during SYMP (*p* = 0.80), and FMD normalized to cumulative shear rate (Fig. 2C) at rest (*p* = 0.09) and during SYMP (*p* = 0.53), were not different between sexes. However, they were affected by SYMP in both sexes (all *p* < 0.0002). Therefore, SYMP-mediated blunting of brachial artery FMD was greater in women compared to men prior to performing any normalization (Fig. 3E; *p* = 0.03), whereas it was no longer different after normalization to baseline brachial artery diameter (Fig. 3F; *p* = 0.11) and shear rate (Fig. 3G; *p* = 0.34). Allometric scaling of FMD (Fig. 2D) also revealed that brachial artery dilation was similar in men compared to women at rest (9.01 ± 3.57% vs 10.25 ± 3.19%, men vs women; *p* = 0.49) and during SYMP (6.43 ± 2.41% vs 5.55 ± 1.98%; *p* = 0.51).

### Hemodynamic data

**Fig. 4:**
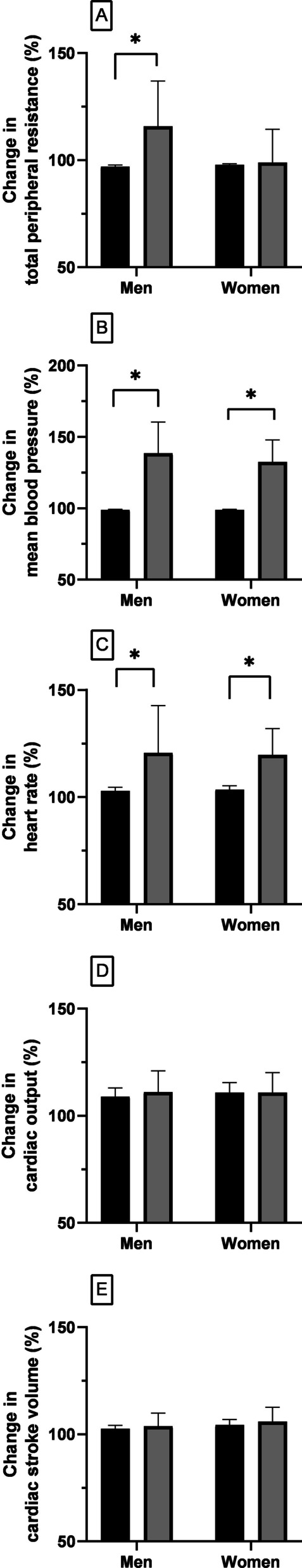
Percent change from preceding baseline values after cuff release in total peripheral resistance (**A**), mean blood pressure (**B**), heart rate (**C**), cardiac output (**D**), and cardiac stroke volume (**E**), at rest (black bars) vs SYMP (grey bars) (*p < 0.05), in men vs women (^#^*p* < 0.05)

TPR over the 60 s after cuff release (Fig. 4A) was augmented during SYMP compared to at rest in men (*p* = 0.004) but not in women (*p* = 0.967). Prior to normalization to baseline values, absolute values of MAP (Additional file 1, Table S18) were similar in women compared to men at rest (*p* = 0.24), but lower in women during SYMP (*p* = 0.005). When the different baseline MAPs between sexes were accounted for, the percent increase from baseline of MAP (Fig. 3B) during SYMP was similar in men compared to women (*p* = 0.436). Moreover, MAP increased in response to SYMP in both sexes (*p* < 0.0001). The heart rate (Fig. 3C) increased in response to SYMP in both sexes (*p* < 0.009) and the percent changes from baseline were similar between sexes (*p* > 0.97). Cardiac output (Fig. 3D) and stroke volume (Fig. 3E) were not affected by SYMP in both sexes (*p* > 0.53) and the percent changes from baseline were similar between sexes (*p* > 0.46).

## Discussion

### Summary and main findings

This study investigated sex differences in the in-vivo forearm VC regulation in young healthy humans. The in-vivo regulation of VC is a continuous balance between sympathetic vasoconstriction and endothelial-mediated vasodilation [10]. The precise regulation of VC is essential for controlling tissue BF and systemic blood pressure [11]. The interaction between sympathetic vasoconstriction and endothelial-mediated vasodilation was evaluated by assessing the sympathetic-mediated blunting of forearm vasodilation after a brief period of ischemia. It was also assessed whether any sympathetic-mediated restriction of vasodilation blunts tissue BF and re-oxygenation after circulatory reperfusion in both sexes. Our data may agree with previous investigations suggesting that women have blunted transduction of sympathetic activity into vasoconstriction compared to men. Indeed, TPR was augmented in response to CPT-induced SYMP in men but not in women. Although the higher brachial artery FMD found in women compared to men may lead to the initial conclusion that women have greater endothelial-mediated vasodilation, this non-normalized result is biased by the smaller baseline brachial artery diameter in women. Indeed, no sex differences in brachial artery FMD were found after normalization to baseline diameter (details below). The percent increases from baseline of peak and average forearm VC after circulatory occlusion, at rest, were similar between sexes. Interestingly, such increases were also similar between sexes during SYMP, regardless of the fact that women showed a blunted increase in TPR in response to SYMP. Thus, as shown in Fig. 3, sympathetic-mediated restriction of vasodilation was similar between sexes. The percent increase from baseline of the average brachial artery BF after cuff release was also similar between sexes at rest and during SYMP. Although SYMP restrained the average forearm VC increment over the 60 s after release of circulatory occlusion, such a restriction did not impair the normal hand re-oxygenation that occurs in that time frame in both sexes.

CPT was used to induce SYMP. CPT is a painful stressor that elevates blood pressure mainly due to peripheral sympathetic vasoconstriction [21]. This stimulant was used in several investigations to assess the sympathetic-mediated blunting of nitric oxide-mediated endothelial vasodilation [18, 19, 22]. The specific timing of application of the CPT stimulus used in our experimental protocol was taken from the study by Dyson et al. [19] to have a direct comparison between previous findings and our own. CPT-induced SYMP has been shown to blunt the normal brachial artery vasodilation upon release of circulatory occlusion [18, 19, 22]. Other non-invasive sympathetic stimulants such as mental arithmetic tasks, lower body suction, and muscle chemoreflex activation failed in this intent [19]. The percent increase from baseline of forearm VC following reperfusion was considered the main research outcome in our study. Increases in VC provide information on the global vasodilation of the forearm, including changes in resistance arterioles and pre-capillary arterioles. Restrictions in the normal forearm VC increase after a brief period of ischemia can potentially affect BF towards the hand and, thus, the hand re-oxygenation in that time frame. The brachial artery FMD was a secondary research outcome in our study. It provides information on nitric oxide-mediated endothelial vasodilation, which has been associated with coronary artery endothelial function [23]. However, brachial artery FMD does not provide information on the precise regulation of BF towards the hand [24]. Hemodynamic changes were calculated via the ModelFlow algorithm of Portapres. This algorithm has been widely validated and used in research [25, 26]. It uses a statistical model of the human circulation to calculate the hemodynamic parameters from the finger arterial pressure waveform.

### Hemodynamic data

Our data show that TPR after cuff release was augmented in men but not in women during CPT-induced SYMP compared to at rest (Fig. 4A). This finding may agree with the results of a previous study that sought to quantify leg sympathetic vasoconstriction in response to CPT in young individuals [17]. CPT induced a blunted decrease in femoral vascular conductance in young women compared to young men, consistent with the notion of a blunted increase in regional peripheral resistance in women [17]. Other studies also showed that sympathetic stimulants induce less vasoconstriction in women compared to men [2]. A blunted increase in TPR in women may agree with the notion that women have blunted transduction of MSNA into vasoconstriction compared to men [2–4]. Indeed, a lack of relationship between MSNA and TPR in women but not in men has been documented [2]. Moreover, women have been suggested to have lower autonomic support of arterial blood pressure control compared to men, as women show a lower arterial pressure drop in response to autonomic blockade compared to men [27]. In our study, men reported higher absolute values of MAP during SYMP compared to women. However, men also reported (not statistically significant) higher values of MAP than women at baseline. After normalization to baseline values, SYMP resulted in similar percent increases of MAP between sexes (Fig. 4B). These results support the notion that the use of absolute values may lead to misleading conclusions if differences in baseline values between sexes are not accounted for. The heart rate was increased during SYMP compared to at rest in both sexes. This finding is consistent with the positive chronotropic effect of sympathetic stimulation on the heart rate [28]. Cardiac output and stroke volume were not affected by SYMP in both sexes. These findings agree with those of previous research showing that cardiac index (cardiac output normalized to body surface area) was unchanged during CPT although TPR increased [18].

### Interaction between vasoconstriction and vasodilation

The precise regulation of VC is essential to regulate BF towards tissues [11]. VC rapidly increases upon release of circulatory occlusion to allow fast blood supply towards the ischemic area. Rapid vasodilation is due to the release of local vasodilator agents produced by the vascular endothelium or muscle itself that quickly relax the vascular smooth muscle [29]. Our data show that the percent increments from baseline of peak (Fig. 1A) and average forearm VC (Fig. 1B) after cuff release were similar between sexes at rest. Brachial artery FMD was also similar between men and women after normalization to baseline brachial artery diameter and shear rate (Fig. 2B–D). Therefore, when baseline values are accounted for, similar forearm VC increments and brachial artery FMDs after cuff release in men compared to women may suggest similar endothelial-mediated vasodilation between sexes. Although women may have a blunted sympathetic vasoconstriction compared to men in response to CPT-induced SYMP, as evinced by the blunted TPR increase in women, the percent increase from baseline of forearm VC after cuff release was similar between sexes during SYMP. Thus, SYMP blunted the normal VC increments (Fig. 3A, B) after cuff release similarly between sexes. These findings suggest that, when comparisons are performed on data normalized to preceding baseline values, there are no overt sex differences in the dynamic regulation of forearm VC assessed through the interaction between endothelial-mediated vasodilation and sympathetic vasoconstriction. SYMP blunted the peak and average forearm VC increase upon cuff release in both sexes. No study has specifically investigated whether SYMP restrains the normal increase in forearm VC; however, SYMP-mediated restriction of vasodilation is consistent with the physiological assumptions. Norepinephrine released from sympathetic nerve endings and adrenal glands during SYMP should oppose the normal nitric oxide-mediated vascular smooth muscle relaxation by binding to post-synaptic α-adrenergic receptors [30].

The percent increments from baseline of peak (Fig. 1C) and average (Fig. 1D) brachial artery BF after cuff release were similar between sexes at rest and during SYMP. Thus, SYMP changed the normal BF responses (Fig. 3C, D) after cuff release similarly between sexes. These results suggest that, when different baseline values of BF between sexes are accounted for, there are no overt sex differences in the dynamic regulation of brachial artery BF towards an ischemic area regardless of a stressful situation. SYMP augmented the percent increase from baseline of peak brachial artery BF in both sexes. This is probably due to the higher limb perfusion pressure (MAP) during SYMP which overpowers the effects of any restrained vasodilation [11]. When comparing changes in absolute values of BF, without taking into account different baseline values between sexes, results may be misleading. Absolute values of peak BF during SYMP compared to at rest resulted to be statistically augmented in men but not in women. This finding may provide further insight into the study by Lind et al. [18], where brachial artery BF immediately after cuff release increased without reaching statistical significance during CPT as compared to without sympathetic stress in a group composed of 10 young men and 8 young women. A blunted change in the absolute peak BF in response to SYMP in women might be responsible for the failure to achieve statistical significance in the previous study. SYMP did not change the percent increment from baseline of the average brachial artery BF over the 60 s after cuff deflation. This suggests that acute levels of sympathetic activation do not impair the normal capacity to provide blood towards an ischemic tissue despite the presence of a blunted forearm vasodilation in both sexes, probably due to a weighted increase of MAP [11].

Hand oxygenation diminished to similar levels after 5 min of forearm cuff occlusion in both sexes, regardless of SYMP (Fig. 1E). Hand oxygenation then increased above preceding baseline levels in both sexes over the 60 s after cuff release, without being impaired by SYMP (Fig. 1F). This suggests that SYMP does not impair the normal hand re-oxygenation after cuff release despite it blunts forearm vasodilation in both sexes. Similar values of hand oxygenation over the 60 s after cuff release during SYMP compared to at rest may be explained by the fact that SYMP does not change the average hyperemic response in that time frame. However, the values of hand oxygenation reached after cuff release were greater in women compared to men. Since the average brachial artery BF after cuff release was similar between sexes, a faster peripheral oxygen extraction dynamic in women compared to men might be involved [31].

Consistent with previous investigations, brachial artery FMD from baseline was higher in women compared to men when expressed as absolute values (Fig. 2A) [8, 32]. However, as previously shown, sex differences were abolished after normalization to baseline diameter [32] and cumulative shear rate (Fig. 2B–D). Women generally have a smaller brachial artery diameter compared to men [8, 32]. The baseline artery diameter has been shown to affect artery FMD, as FMD is higher in smaller arteries due to the higher shear rate during reactive hyperemia, and vice versa [8, 15]. In our study, SYMP blunted the brachial artery FMD in both sexes. This is in agreement with previous research [18, 19] which, however, did not investigate sex differences. In this regard, our data show that FMD attenuation by SYMP was higher in women compared to men when expressed as absolute values (Fig. 3E). Previous studies suggested a sex-related sensitivity in the regulation of large-artery vascular tone, as evinced by more pronounced shear-mediated arterial vasodilation and vasoconstriction in women compared to men [33]. Consistent with this notion, sex differences in brachial artery FMD attenuation were abolished after normalization to shear rate and baseline diameter (Fig. 3F, G).

## Limitations

This study did not aim to provide the physiological mechanisms underlying sex differences. As previously done in similar studies [22, 34], we did not measure MSNA, cortisol, epinephrine, norepinephrine, or oestrogen. Evaluation of these variables could provide further clarification regarding sex differences in neurovascular modulation. Using the analogous experimental protocol employed in our project, other researchers showed that CPT increases only blood norepinephrine by 1 min after cuff deflation in young men, without affecting blood epinephrine and serum cortisol [19]. Other studies, however, have suggested that some variables change immediately after the stress application, whereas others show delayed responses, such as peak cortisol concentration [34].

## Perspectives and significance

Neurovascular regulation is based on the continuous interaction between sympathetic vasoconstriction and endothelial-mediated vasodilation. However, the current understanding of sex differences in neurovascular regulation is predominantly based on studies in which these two aspects have been investigated separately. The lack of knowledge of how these two aspects interact could lead to apparently rational, but erroneous, speculations about neurovascular regulation in-vivo, such as the hypothesis we tested that men had greater sympathetic blunting of endothelial-mediated vasodilation. Our data normalized to baseline values show similar sympathetic-mediated restriction of vasodilation between sexes. This suggests that speculations on how the dynamic neurovascular regulation differs between sexes cannot be based upon “mathematical operations” between vasodilation and vasoconstriction differences assessed separately. Furthermore, previous similar investigations have focused on the sympathetic-mediated blunting of FMD of the brachial artery. This variable is related to cardiovascular risk but is not a key regulator of brachial artery BF and blood pressure. Therefore, the results of our study encourage investigating sex differences in the interaction between sympathetic vasoconstriction and endothelial-mediated vasodilation on VC, as well as how this integrated regulation eventually affects tissue BF and blood pressure.

## Conclusions

When considered separately, women show similar endothelial vasodilation compared to men, as well as a likely blunted sympathetic vasoconstriction in response to CPT-induced SYMP. However, the interaction between vasoconstriction and vasodilation leads to a similar regulation of forearm VC between sexes. Indeed, sympathetic-mediated restriction of vasodilation is similar between sexes. Although SYMP restrains the normal forearm VC increase in both sexes, SYMP does not impair the normal hyperemic response or the normal hand re-oxygenation after a brief period of ischemia in both sexes.

## Supplementary Information

**Additional file 1.** ANOVA results (effect of sex; effect of SYMP; interaction), numerical values of the data, and posthoc test results (Rest vs SYMP; Men vs Women).

## Data Availability

The data sets supporting the conclusions of this article are included within the article and its Additional file 1.

## Abbreviations

BF: Blood flow
CPT: Cold pressor test
FMD: Flow-mediated dilation
MAP: Mean arterial pressure
MSNA: Muscle sympathetic nerve activity
SYMP: Acute sympathetic activation
TPR: Total peripheral resistance
VC: Vascular conductance
