# Supplementary material for: Sympathetic-mediated blunting of forearm vasodilation is similar between young men and women
Source: Biol Sex Differ. 2022 Jun 25;13:33. doi: 10.1186/s13293-022-00444-0 (PMC9233837; doi:10.1186/s13293-022-00444-0)
Supplement: Supplementary file 1 — Additional file 1. ANOVA results (effect of sex; effect of SYMP; interaction), numerical values of the data, and posthoc test results (Rest vs SYMP; Men vs Women). [file 13293_2022_444_MOESM1_ESM.docx]

| Table S1. ANOVA results (effect of sex; effect of SYMP; interaction), numerical values of the data, and posthoc test results (Rest vs SYMP; Men vs Women) of the percent increase from baseline of peak vascular conductance | | | |
| --- | --- | --- | --- |
| ANOVA | F-value | P-value |  |
| Sex | F (1. 23) = 0.001640 | P=0.9680 |  |
| SYMP | F (1. 23) = 35.69 | P<0.0001 |  |
| Interaction | F (1. 23) = 0.6755 | P=0.4196 |  |
| (%) | Men | Women | Men vs Women |
| Rest | 911±248 | 923±282 | P=0.99 |
| SYMP | 811±244 | 791±268 | P=0.98 |
| Rest vs SYMP | P=0.003 | P=0.0001 |  |

| Table S2. ANOVA results (effect of sex; effect of SYMP; interaction), numerical values of the data, and posthoc test results (Rest vs SYMP; Men vs Women) of the absolute values of peak vascular conductance | | | |
| --- | --- | --- | --- |
| ANOVA | F-value | P-value |  |
| Sex | F (1. 22) = 25.02 | P=0.0015 |  |
| SYMP | F (1. 22) = 26.34 | P<0.0001 |  |
| Interaction | F (1. 22) = 0.2094 | P=0.8039 |  |
| (mL/mmHg) | Men | Women | Men vs Women |
| Rest | 8.86±2.96 | 5.51±1.38 | P=0.002 |
| SYMP | 7.93±3.23 | 4.66±1.20 | P=0.002 |
| Rest vs SYMP | P=0.003 | P=0.004 |  |

| Table S3. ANOVA results (effect of sex; effect of SYMP; interaction), numerical values of the data, and posthoc test results (Rest vs SYMP; Men vs Women) of the percent increase from baseline of average vascular conductance | | | |
| --- | --- | --- | --- |
| ANOVA | F-value | P-value |  |
| Sex | F (1. 23) = 0.04176 | P=0.8399 |  |
| SYMP | F (1. 23) = 98.89 | P<0.0001 |  |
| Interaction | F (1. 23) = 0.005987 | P=0.9390 |  |
| (%) | Men | Women | Men vs Women |
| Rest | 508±129 | 501±128 | P=0.98 |
| SYMP | 312±90 | 322±96 | P=0.97 |
| Rest vs SYMP | P<0.0001 | P<0.0001 |  |

| Table S4. ANOVA results (effect of sex; effect of SYMP; interaction), numerical values of the data, and posthoc test results (Rest vs SYMP; Men vs Women) of the absolute values of average vascular conductance | | | |
| --- | --- | --- | --- |
| ANOVA | F-value | P-value |  |
| Sex | F (1. 23) = 10.84 | P=0.0032 |  |
| SYMP | F (1. 23) = 63.98 | P<0.0001 |  |
| Interaction | F (1. 23) = 2.763 | P=0.1100 |  |
| (mL/mmHg) | Men | Women | Men vs Women |
| Rest | 5.77±2.30 | 3.47±1.18 | P=0.001 |
| SYMP | 3.80±1.77 | 2.17±0.61 | P=0.02 |
| Rest vs SYMP | P<0.0001 | P=0.0003 |  |

| Table S5. ANOVA results (effect of sex; effect of SYMP; interaction), numerical values of the data, and posthoc test results (Rest vs SYMP; Men vs Women) of the percent increase from baseline of peak blood flow | | | |
| --- | --- | --- | --- |
| ANOVA | F-value | P-value |  |
| Sex | F (1. 23) = 0.003655 | P=0.9523 |  |
| SYMP | F (1. 23) = 23.36 | P<0.0001 |  |
| Interaction | F (1. 23) = 1.386 | P=0.2511 |  |
| (%) | Men | Women | Men vs Women |
| Rest | 909±247 | 977±335 | P=0.87 |
| SYMP | 1213±369 | 1162±426 | P=0.92 |
| Rest vs SYMP | P=0.0007 | P=0.0291 |  |

| Table S6. ANOVA results (effect of sex; effect of SYMP; interaction), numerical values of the data, and posthoc test results (Rest vs SYMP; Men vs Women) of the absolute values of peak blood flow | | | |
| --- | --- | --- | --- |
| ANOVA | F-value | P-value |  |
| Sex | F (1. 22) = 25 | P=0.001 |  |
| SYMP | F (1. 22) = 12 | P=0.003 |  |
| Interaction | F (1. 22) = 3.5 | P=0.096 |  |
| (mL) | Men | Women | Men vs Women |
| Rest | 711±232 | 431±105 | P=0.01 |
| SYMP | 983±490 | 516±175 | P<0.001 |
| Rest vs SYMP | P=0.004 | P=0.46 |  |

| Table S7. ANOVA results (effect of sex; effect of SYMP; interaction), numerical values of the data, and posthoc test results (Rest vs SYMP; Men vs Women) of the percent increase from baseline of average blood flow | | | |
| --- | --- | --- | --- |
| ANOVA | F-value | P-value |  |
| Sex | F (1. 23) = 0.1238 | P=0.7282 |  |
| SYMP | F (1. 23) = 5.323 | P=0.0304 |  |
| Interaction | F (1. 23) = 0.3942 | P=0.5363 |  |
| (%) | Men | Women | Men vs Women |
| Rest | 554±149 | 551±162 | P=0.99 |
| SYMP | 508±161 | 471±175 | P=0.80 |
| Rest vs SYMP | P=0.45 | P=0.09 |  |

| Table S8. ANOVA results (effect of sex; effect of SYMP; interaction), numerical values of the data, and posthoc test results (Rest vs SYMP; Men vs Women) of the absolute values of average blood flow | | | |
| --- | --- | --- | --- |
| ANOVA | F-value | P-value |  |
| Sex | F (1. 23) = 13 | P=0.001 |  |
| SYMP | F (1. 23) = 2.5 | P=0.126 |  |
| Interaction | F (1. 23) = 0.032 | P=0.859 |  |
| (mL) | Men | Women | Men vs Women |
| Rest | 446±180 | 251±80 | P=0.004 |
| SYMP | 415±215 | 212±68 | P=0.002 |
| Rest vs SYMP | P=0.56 | P=0.38 |  |

| Table S9. ANOVA results (effect of sex; effect of SYMP; interaction), numerical values of the data, and posthoc test results (Rest vs SYMP; Men vs Women) of the percent increase from baseline of hand oxygenation after cuff inflation | | | |
| --- | --- | --- | --- |
| ANOVA | F-value | P-value |  |
| Sex | F (1. 23) = 0.1571 | P=0.6955 |  |
| SYMP | F (1. 23) = 2.075 | P=0.1632 |  |
| Interaction | F (1. 23) = 0.3893 | P=0.5388 |  |
| (%) | Men | Women | Men vs Women |
| Rest | 65.9±12.6 | 67.0±9.3 | P=0.97 |
| SYMP | 66.9±14.4 | 69.4±10.2 | P=0.83 |
| Rest vs SYMP | P=0.82 | P=0.28 |  |

| Table S10. ANOVA results (effect of sex; effect of SYMP; interaction), numerical values of the data, and posthoc test results (Rest vs SYMP; Men vs Women) of the percent increase from baseline of hand oxygenation after cuff release | | | |
| --- | --- | --- | --- |
| ANOVA | F-value | P-value |  |
| Sex | F (1. 23) = 10.53 | P=0.0036 |  |
| SYMP | F (1. 23) = 8.249 | P=0.0086 |  |
| Interaction | F (1. 23) = 0.06653 | P=0.7987 |  |
| (%) | Men | Women | Men vs Women |
| Rest | 105.4±2.8 | 115.2±9.9 | P=0.01 |
| SYMP | 110.9±6.8 | 119.8±11.3 | P=0.02 |
| Rest vs SYMP | P=0.08 | P=0.14 |  |

| Table S11. ANOVA results (effect of sex; effect of SYMP; interaction), numerical values of the data, and posthoc test results (Rest vs SYMP; Men vs Women) of the brachial artery diameter FMD | | | |
| --- | --- | --- | --- |
| ANOVA | F-value | P-value |  |
| Sex | F (1. 23) = 5.072 | P=0.0342 |  |
| SYMP | F (1. 23) = 72.74 | P<0.0001 |  |
| Interaction | F (1. 23) = 5.205 | P=0.0321 |  |
| (%) | Men | Women | Men vs Women |
| Rest | 4.96±2.07 | 7.27±2.36 | P=0.016 |
| SYMP | 3.61±1.99 | 4.93±1.88 | P=0.23 |
| Rest vs SYMP | P=0.0005 | P<0.0001 |  |

| Table S12. ANOVA results (effect of sex; effect of SYMP; interaction), numerical values of the data, and posthoc test results (Rest vs SYMP; Men vs Women) of the peak shear rate | | | |
| --- | --- | --- | --- |
| ANOVA | F-value | P-value |  |
| Sex | F (1. 23) = 2.610 | P=0.1198 |  |
| SYMP | F (1. 23) = 179.6 | P<0.0001 |  |
| Interaction | F (1. 23) = 0.1012 | P=0.7532 |  |
| (1/s) | Men | Women | Men vs Women |
| Rest | 2057±547 | 2471±619 | P=0.38 |
| SYMP | 4152±901 | 4669±1093 | P=0.23 |
| Rest vs SYMP | P<0.0001 | P<0.0001 |  |

| Table S13. ANOVA results (effect of sex; effect of SYMP; interaction), numerical values of the data, and posthoc test results (Rest vs SYMP; Men vs Women) of the cumulative (AUC) shear rate | | | |
| --- | --- | --- | --- |
| ANOVA | F-value | P-value |  |
| Sex | F (1. 23) = 0.8341 | P=0.3706 |  |
| SYMP | F (1. 23) = 45.77 | P<0.0001 |  |
| Interaction | F (1. 23) = 0.0002686 | P=0.9871 |  |
| (s^-1^x60s) | Men | Women | Men vs Women |
| Rest | 79K±26K | 88K±23K | P=0.65 |
| SYMP | 109K±29K | 118K±30K | P=0.65 |
| Rest vs SYMP | P=0.0002 | P=0.0001 |  |

| Table S14. ANOVA results (effect of sex; effect of SYMP; interaction), numerical values of the data, and posthoc test results (Rest vs SYMP; Men vs Women) of the brachial artery FMD normalized to cumulative shear rate | | | |
| --- | --- | --- | --- |
| ANOVA | F-value | P-value |  |
| Sex | F (1. 23) = 3.294 | P=0.0826 |  |
| SYMP | F (1. 23) = 70.59 | P<0.0001 |  |
| Interaction | F (1. 23) = 0.9579 | P=0.3379 |  |
| (e^-005^ %/s^-1^x60s) | Men | Women | Men vs Women |
| Rest | 6.70±3.01 | 8.47±2.46 | P=0.09 |
| SYMP | 3.30±1.52 | 4.17±1.09 | P=0.53 |
| Rest vs SYMP | P<0.0001 | P<0.0001 |  |

| Table S15. ANOVA results (effect of sex; effect of SYMP; interaction), numerical values of the data, and posthoc test results (Rest vs SYMP; Men vs Women) of the brachial artery FMD normalized to baseline diameter | | | |
| --- | --- | --- | --- |
| ANOVA | F-value | P-value |  |
| Sex | F (1. 23) = 1.421 | P=0.2454 |  |
| SYMP | F (1. 23) = 83.71 | P<0.0001 |  |
| Interaction | F (1. 23) = 2.718 | P=0.1128 |  |
| (% x cm) | Men | Women | Men vs Women |
| Rest | 1.84±0.49 | 2.18±0.53 | P=0.20 |
| SYMP | 1.35±0.59 | 1.47±0.41 | P=0.80 |
| Rest vs SYMP | P<0.0001 | P<0.0001 |  |

| Table S16. ANOVA results (effect of sex; effect of SYMP; interaction), numerical values of the data, and posthoc test results (Rest vs SYMP; Men vs Women) of the percent change from baseline of total peripheral resistance | | | |
| --- | --- | --- | --- |
| ANOVA | F-value | P-value |  |
| Sex | F (1. 23) = 4.9 | P=0.036 |  |
| SYMP | F (1. 23) = 7.2 | P=0.013 |  |
| Interaction | F (1. 23) = 5.6 | P=0.027 |  |
| (%) | Men | Women | Men vs Women |
| Rest | 97.07±0.61 | 97.79±0.54 | P=0.988 |
| SYMP | 115.83±21.1 | 98.99±15.4 | P=0.004 |
| Rest vs SYMP | P=0.004 | P=0.967 |  |

| Table S17. ANOVA results (effect of sex; effect of SYMP; interaction), numerical values of the data, and posthoc test results (Rest vs SYMP; Men vs Women) of the percent change from baseline of mean arterial pressure | | | |
| --- | --- | --- | --- |
| ANOVA | F-value | P-value |  |
| Sex | F (1. 23) = 0.70 | P=0.410 |  |
| SYMP | F (1. 23) = 94 | P<0.0001 |  |
| Interaction | F (1. 23) = 0.66 | P=0.425 |  |
| (%) | Men | Women | Men vs Women |
| Rest | 98.90±0.45 | 98.84±0.40 | P=0.99 |
| SYMP | 138.62±21.85 | 132.43±15.32 | P=0.436 |
| Rest vs SYMP | P<0.0001 | P<0.0001 |  |

| Table S18. ANOVA results (effect of sex; effect of SYMP; interaction), numerical values of the data, and posthoc test results (Rest vs SYMP; Men vs Women) of the absolute values of mean blood pressure | | | |
| --- | --- | --- | --- |
| ANOVA | F-value | P-value |  |
| Sex | F (1. 23) = 10.91 | P=0.0031 |  |
| SYMP | F (1. 23) = 131.5 | P<0.0001 |  |
| Interaction | F (1. 23) = 1.433 | P=0.2434 |  |
| (mmHg) | Men | Women | Men vs Women |
| Rest | 78.1±8.7 | 72.8±6.7 | P=0.24 |
| SYMP | 108.0±8.3 | 97.1±9.9 | P=0.005 |
| Rest vs SYMP | P<0.0001 | P<0.0001 |  |

| Table S19. ANOVA results (effect of sex; effect of SYMP; interaction), numerical values of the data, and posthoc test results (Rest vs SYMP; Men vs Women) of the percent change from baseline of heart rate | | | |
| --- | --- | --- | --- |
| ANOVA | F-value | P-value |  |
| Sex | F (1. 23) = 0.00072 | P=0.979 |  |
| SYMP | F (1. 23) = 23 | P<0.001 |  |
| Interaction | F (1. 23) = 0.049 | P=0.826 |  |
| (%) | Men | Women | Men vs Women |
| Rest | 102.85±1.79 | 103.55±1.78 | P=0.988 |
| SYMP | 120.62±22.15 | 119.73±12.24 | P=0.981 |
| Rest vs SYMP | P=0.004 | P=0.007 |  |

| Table S20. ANOVA results (effect of sex; effect of SYMP; interaction), numerical values of the data, and posthoc test results (Rest vs SYMP; Men vs Women) of the absolute values of heart rate | | | |
| --- | --- | --- | --- |
| ANOVA | F-value | P-value |  |
| Sex | F (1. 23) = 0.02683 | P=0.8713 |  |
| SYMP | F (1. 23) = 20.78 | P=0.0001 |  |
| Interaction | F (1. 23) = 0.03968 | P=0.8439 |  |
| (bpm) | Men | Women | Men vs Women |
| Rest | 68.2±6.4 | 67.8±16.3 | P=0.99 |
| SYMP | 79.3±11.8 | 78.0±18.7 | P=0.97 |
| Rest vs SYMP | P=0.0063 | P=0.009 |  |

| Table S21. ANOVA results (effect of sex; effect of SYMP; interaction), numerical values of the data, and posthoc test results (Rest vs SYMP; Men vs Women) of the percent change from baseline of cardiac output | | | |
| --- | --- | --- | --- |
| ANOVA | F-value | P-value |  |
| Sex | F (1. 23) = 0.11 | P=0.745 |  |
| SYMP | F (1. 23) = 0.47 | P=0.498 |  |
| Interaction | F (1. 23) = 0.37 | P=0.548 |  |
| (%) | Men | Women | Men vs Women |
| Rest | 108.95±4.10 | 110.78±4.68 | P=0.61 |
| SYMP | 111.14±9.91 | 110.91 ±9.24 | P=0.99 |
| Rest vs Symp | P=0.79 | P=0.99 |  |

| Table S22. ANOVA results (effect of sex; effect of SYMP; interaction), numerical values of the data, and posthoc test results (Rest vs SYMP; Men vs Women) of the percent change from baseline of cardiac stroke volume | | | |
| --- | --- | --- | --- |
| ANOVA | F-value | P-value |  |
| Sex | F (1. 23) = 1.6 | P=0.224 |  |
| SYMP | F (1. 23) = 1.6 | P=0.225 |  |
| Interaction | F (1. 23) = 0.035 | P=0.852 |  |
| (%) | Men | Women | Men vs Women |
| Rest | 102.66±1.51 | 104.41±2.52 | P=0.60 |
| SYMP | 103.82±6.08 | 105.97 ±6.65 | P=0.46 |
| Rest vs SYMP | P=0.72 | P=0.53 |  |
